# Data Notes: A New Article Type for Preserving and Sharing Biodiversity Data

**DOI:** 10.1002/ece3.73345

**Published:** 2026-05-17

**Authors:** Bruno E. Soares, Gareth B. Jenkins

**Affiliations:** ^1^ Institute of Environmental Change & Society University of Regina Regina Canada; ^2^ Programa de Pós‐Graduação Em Ecologia Universidade Federal Do Rio de Janeiro Rio de Janeiro Brazil; ^3^ John Wiley & Sons Ltd. Oxford UK

## Abstract

Ecological datasets are frequently at risk of loss due to poor curation and lack of long‐term preservation strategies. To address this challenge, *Ecology and Evolution* is launching Data Notes, a new article category that provides a citable home for well‐documented datasets. Data Notes require structured descriptions of sampling design, metadata, and curation procedures, improving discoverability and reusability. By promoting the archiving of datasets in trusted repositories, this new category aims to strengthen the long‐term accessibility of valuable biodiversity data.

*Ecology and Evolution's* guiding philosophy is to be “author‐friendly.” We seek to remove obstacles to publication and assessing research on the basis of methodological rigor (i.e., sound science; Moore 2011), ensuring that valuable contributions in ecology, evolutionary biology and conservation science have a home in the scientific literature, by disregarding the subjective criterion of novelty as a barrier. We also like to adopt new article categories in response to what our authors need. Over the years, we have introduced new article types, such as Academic Practice in *Ecology and Evolution* (Moore et al. 2017), Nature Notes (Moore et al. 2020), Genetics Notes (Jenkins et al. 2024), greatly expanding the scope of articles we publish in response to scientific needs not fully met by the conventional formats. Today, we are expanding this vision once again by launching a new category: Data Notes.

## 1 Why Data Notes, and Why Now?

High‐quality datasets are essential for advancing research in ecology, evolution, conservation, and environmental science. They define ecological baselines, reveal biodiversity and environmental change, support the synthesis of information to solidify knowledge, and guide decision‐making. Despite their importance, large amounts of ecological and biodiversity data remain poorly curated, inaccessible, or at risk of permanent loss, often existing only in personal computers, paper notebooks, outdated media, or undocumented digital files. For example, an effort to recover datasets from projects funded in the aftermath of the Exxon Valdez oil spill found that 70% of datasets were unrecoverable and are now effectively nonexistent (Jones et al. 2018). Assessing 516 articles using morphometrics data, Vines et al. (2014) showed that the probability of a given dataset being available decreased by 17% every year since publication, demonstrating that self‐archiving does not reliably conserve research data. Even when published in scientific articles, the information might be incomplete or archived in ways that hamper their further usage (Roche et al. 2015).

These are not isolated cases and, as a result, entire lines of evidence on ecological, evolutionary, and environmental research risk being lost, weakening our collective ability to understand, predict, and mitigate biodiversity change. Without intentional preservation and open archiving, even publicly funded datasets can disappear within a couple of years. Data Notes aim to directly address this problem, creating a dedicated space for preserving and sharing these data in a structured, transparent way. We aim to ensure long‐term preservation of datasets in trusted repositories, improve data completeness and quality in available information, increase discoverability and reuse, create a venue to credit the labor behind such data creation and curation, and support open science practices grounded in FAIR and CARE principles.

## 2 What Are Data Notes?

Data Notes are short format articles dedicated to describing curated datasets of value to the ecology and evolution community. They provide a permanent version of record, and a citable home for datasets that are well‐documented; they require detailed explanation of sampling design, metadata structure, or curation processes. Such materials can represent a wide variety of data of interest for the community, such as but not limited to: historical datasets or long‐term monitoring; individual‐level information on prey–predator interactions; morphological, physiological, or behavioral trait measurements; GPS and telemetry records for animals. Data Notes will be evaluated on methodological soundness, completeness, documentation quality, and anticipated utility, not on novelty.

## 3 Recommended Structure

We are establishing a guiding structure to ensure transparency, accessibility, and long‐term scientific value, which is recommended, but not mandated. We recommend submitted materials to include the following components:

– Background: Context, purpose, and scientific relevance of the dataset; history of collection.
– Data Collection and Curation Procedures: Sampling design, methodology, data compilation, metadata creation, and curation steps.
– Summary Information: Description of datasets, tables, variables, file formats (open, non‐proprietary), naming conventions, taxonomic and spatial coverage, and data organization principles.
– Usage Notes: Guidance for reuse, limitations, caveats, and recommendations for integration with other datasets.

## 4 Preservation, Metadata, and Open Repositories

Data Notes must follow *Ecology and Evolution's* open data policy (Jenkins et al. 2023) and comply with FAIR principles for Findability, Accessibility, Interoperability, and Reusability. Datasets must be archived in trustworthy repositories (e.g., Dryad, Zenodo, GBIF, OSF, Figshare, domain‐specific repositories), using open formats (CSV, TIFF, TXT, etc.), appropriately structured metadata, ideally both human‐ and machine‐readable, stable DOIs, and clear licensing (e.g., CC‐BY).

## 5 Toward a Culture of Data Preservation

Launching Data Notes is both a practical and symbolic step toward strengthening the culture of open and responsible data stewardship in ecological and evolutionary research. Data rescue efforts show that intentional preservation, clear metadata, and community‐supported archiving practices support data longevity. Without these intentional efforts, we risk losing biodiversity information that is irreplaceable and could allow us to understand and preserve nature. Our goal is to work with authors to ensure that valuable datasets remain accessible, reusable, and preserved for generations to come.

We invite researchers to submit Data Notes and to help shape this new article type as it evolves. As with previous launches, we will continue to refine this category with input from authors, reviewers, societies, and partners in the open data community. We look forward to seeing your contributions, and to strengthening together the foundations of biodiversity science.

## Author Contributions

**Bruno E. Soares:** conceptualization (equal), writing – original draft (lead). **Gareth B. Jenkins:** conceptualization (equal), writing – review and editing (lead).

## Conflicts of Interest

The authors declare no conflicts of interest.

## Data Availability

No data was created or analyzed in this manuscript.
